# Supplementary material for: Sexual-dimorphism in human immune system aging
Source: Nat Commun. 2020 Feb 6;11:751. doi: 10.1038/s41467-020-14396-9 (PMC7005316; doi:10.1038/s41467-020-14396-9)
Supplement: Supplementary file 1 — Supplementary information [file 41467_2020_14396_MOESM1_ESM.pdf]

Marquez et al., Sexual-dimorphism in human immune system aging.

Supplementary Figures.

Supplementary Figure 1. Age is the main driver of variation in PBMC genomic data.

Supplementary Figure 2. Shared and sex-specific epigenomic signatures of aging.

Supplementary Figure 3. Chromatin accessibility profiles for example loci.

Supplementary Figure 4. Shared and sex-specific transcriptomic signatures.

Supplementary Figure 5. Sex-dimorphic changes in monocyte- and B cell-associated loci.

Supplementary Figure 6. Epigenome and transcriptome changes over human adult lifespan.

Supplementary Figure 7. Sex-specific patterns in temporal peaks/genes.

Supplementary Figure 8. Genomic differences between sexes at different ages.

Figure S1

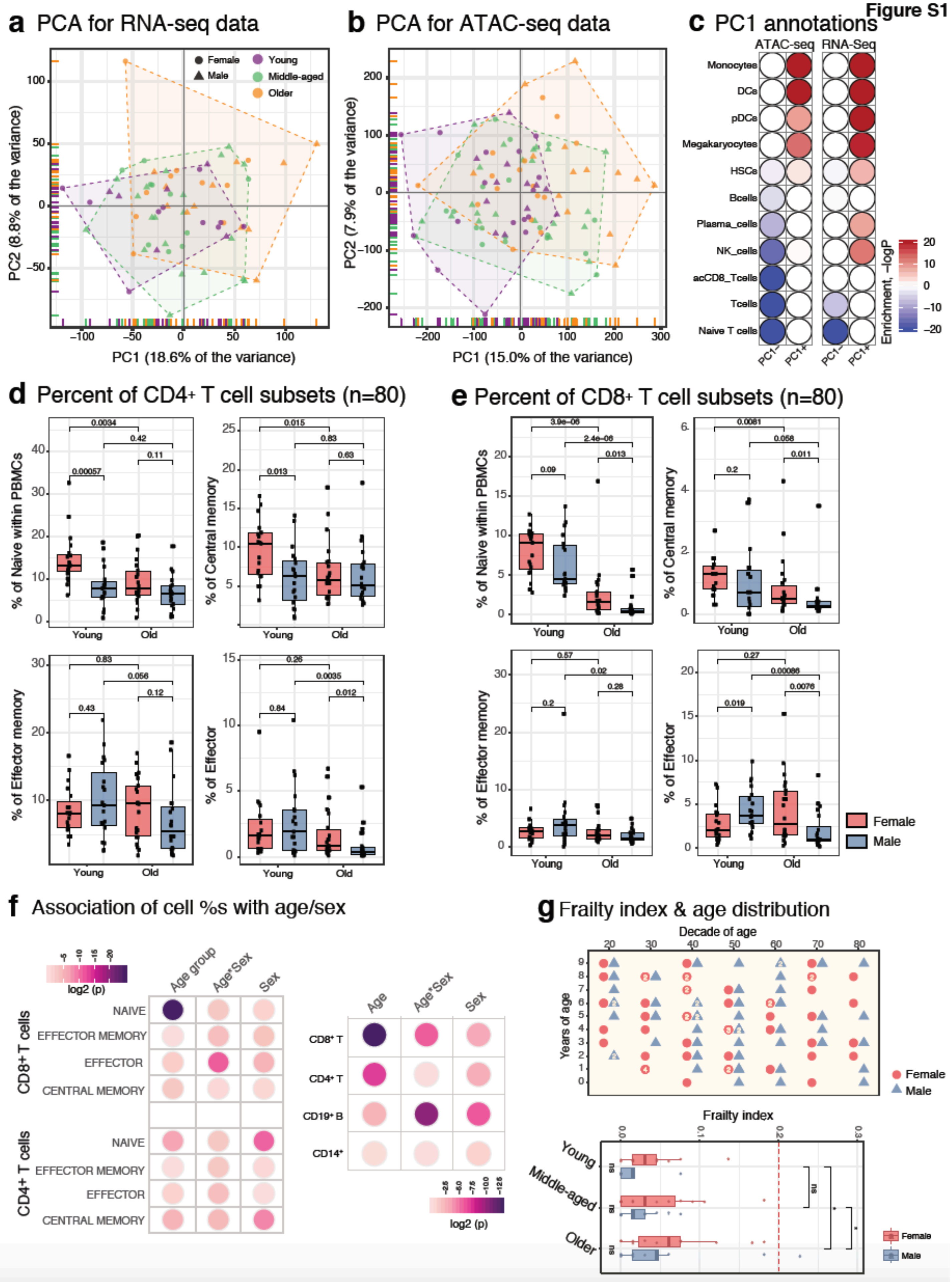
a PCA for RNA-seq data
Female
Male
Young
Middle-aged
Older
PC1 (18.6% of the variance)
PC2 (8.8% of the variance)
b PCA for ATAC-seq data
PC1 (15.0% of the variance)
PC2 (7.9% of the variance)
c PC1 annotations
ATAC-seq
RNA-Seq
Monocytes
DCs
pDCs
Megakaryocytes
HSCs
Bcells
Plasma_cells
NK_cells
acCD8_Tcells
Tcells
Naive T cells
PC1−
PC1+
Enrichment, −logP
d Percent of CD4+ T cell subsets (n=80)
% of Naive within PBMCs
% of Central memory
% of Effector memory
% of Effector
Young
Old
e Percent of CD8+ T cell subsets (n=80)
Female
Male
f Association of cell %s with age/sex
log2 (p)
Age group
Age*Sex
Sex
CD8+ T cells
CD4+ T cells
NAIVE
EFFECTOR MEMORY
EFFECTOR
CENTRAL MEMORY
Age
CD8+ T
CD4+ T
CD19+ B
CD14+
g Frailty index & age distribution
Decade of age
Years of age
Frailty index
Young
Middle-aged
Older
ns

Supplementary Figure 1. **Age is the main driver of variation in PBMC genomic data.** Principal component analyses for (**a**) RNA-seq (n=74) and (**b**) ATAC-seq samples (n=100). PC1 explains 18.6% of variation in RNA-seq, 15% of variation in ATAC-seq data. (**c**) Functional enrichment of PC1-related genes using gene sets obtained from PBMC single-cell RNA-seq data. Marker genes are selected using PC1 scores that are >= 25th percentile: top (positive) and bottom (negative). Genes with high and positive scores were enriched in myeloid cells (monocytes, DCs), whereas genes with high and negative scores were associated to adaptive cells, particularly naïve T cells. These enrichments align well with age-related increase in myeloid lineage and age-related decline in naïve T cell activity. Hypergeometric test is used to calculate enrichment p values. (**d-e**) Flow cytometry data (n= 80) reflect proportions of $CD4^+$ (**d**) and $CD8^+$ (**e**) T cell subsets within PBMCs. Subjects are stratified based on age groups and sex: Young: 22-40, Old: 65+ years old. Wilcoxon signed rank (two-sided) test is used to compare data from men and women, old and young subjects. Naïve $CD4^+$ and $CD8^+$ T cell proportions decline significantly with age in both sexes. (**f**) Significance values from generalized linear models (GLM) to establish the association between PBMC cell composition data with age group, sex, and their interaction (age*sex). Darker colors indicate stronger association. Age is strongly associated with the variation in naïve T cell proportions, whereas sex, age*sex is strongly associated with the variation in B cell proportions. (**g**) Top: Age of subjects spread comparable between sexes across decades. Bottom: Distribution of Rockwood frailty index (FI) values[39] stratified by age groups and sexes. No significant difference detected between sexes for age groups. The FI values increase with age, more so in women with none of the individuals having a value greater than 0.25. Most subjects were non-frail (FI: 0-0.1), a few were prefrail (FI >0.1-0.21) but regardless the frailty did not influence the observed genomic changes. Boxplots represent median, upper and lower quartiles. Source data are provided as a Source Data file for panels c, g.

Figure S2

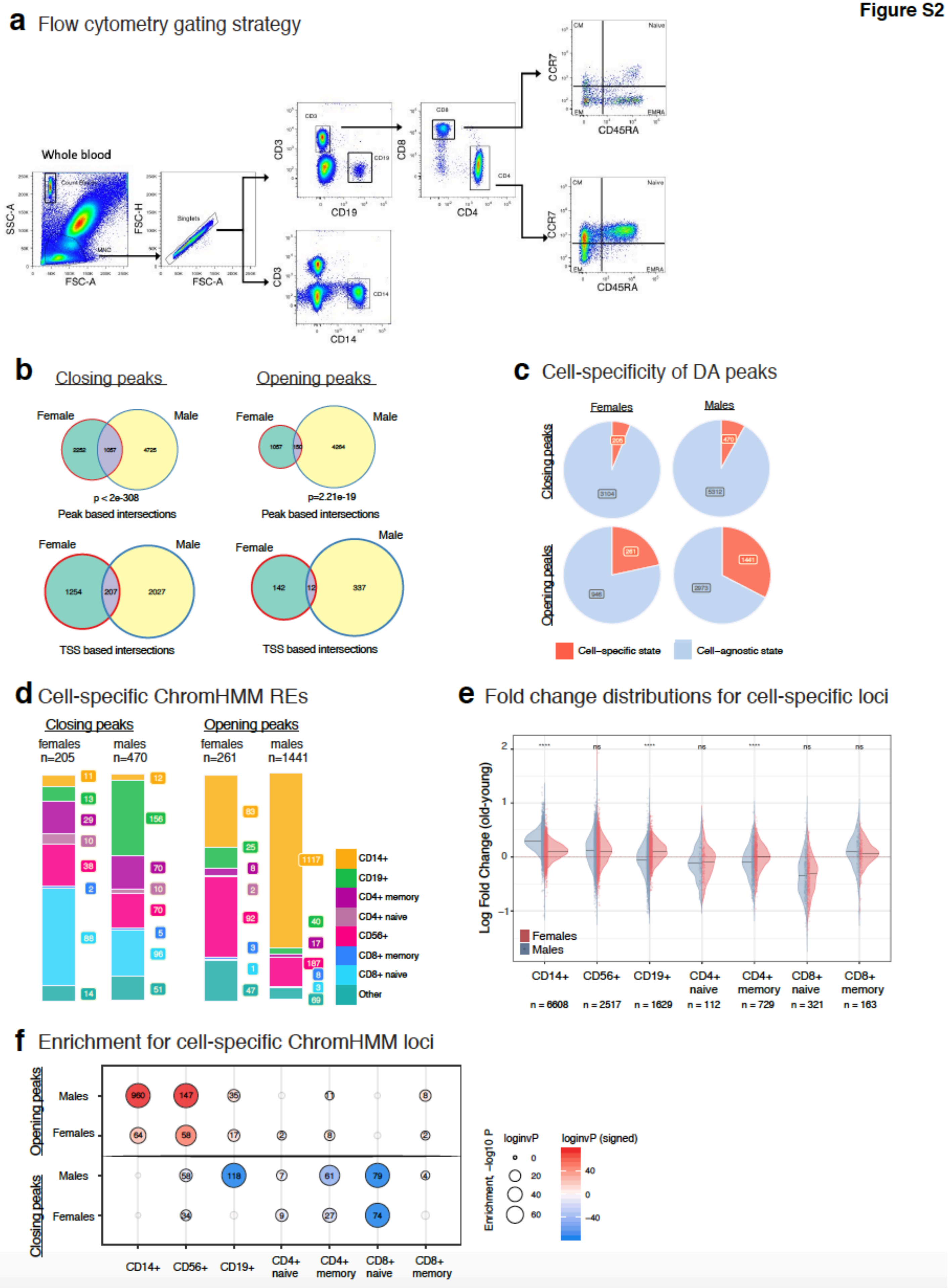
a Flow cytometry gating strategy
Whole blood
SSC-A
FSC-A
FSC-H
Singlets
MNC
CD3
CD19
CD8
CD4
CD14
CCR7
CD45RA
CM
Naive
EM
EMRA
b Closing peaks
Opening peaks
Female
Male
2252
1067
4725
1057
150
4264
p < 2e-308
p=2.21e-19
Peak based intersections
1254
207
2027
142
12
337
TSS based intersections
c Cell-specificity of DA peaks
Females
Males
Closing peaks
Opening peaks
205
3104
470
5312
261
946
1441
2973
Cell-specific state
Cell-agnostic state
d Cell-specific ChromHMM REs
Closing peaks
Opening peaks
females n=205
males n=470
females n=261
males n=1441
CD14+
CD19+
CD4+ memory
CD4+ naive
CD56+
CD8+ memory
CD8+ naive
Other
e Fold change distributions for cell-specific loci
Log Fold Change (old-young)
Females
Males
CD14+ n = 6608
CD56+ n = 2517
CD19+ n = 1629
CD4+ naive n = 112
CD4+ memory n = 729
CD8+ naive n = 321
CD8+ memory n = 163
f Enrichment for cell-specific ChromHMM loci
Opening peaks
Closing peaks
Males
Females
CD14+
CD56+
CD19+
CD4+ naive
CD4+ memory
CD8+ naive
CD8+ memory
Enrichment, −log10 P
loginvP
loginvP (signed)

Supplementary Figure 2. Shared and sex-specific epigenomic signatures of aging. (**a**) Gating strategy used in flow cytometry data generation (**b**) Overlap between differentially accessible (DA) peaks (top) and genes associated to DA peaks (bottom) in women and men. Closing peaks overlap more between men and women. Note that the overlap is statistically significant for both opening and closing peaks based on Fisher's exact test for p-value calculations. (**c**) Distribution of cell-specific and cell-agnostic loci among DA peaks based on chromHMM states in PBMCs and immune cell subsets. Cell-specific regions are notably more common among opening peaks. (**d**) Number of cell-specific loci among closing/opening peaks in men and women. T and B cell-specific peaks are more prevalent among closing peaks, whereas NK and $CD14^+$ monocyte specific peaks are more prevalent among opening peaks. (**e**) Fold change (old-young) distributions for chromatin accessibility levels at cell-specific loci. Biggest decrease is observed in naïve $CD8^+$ T cell-specific loci in both men and women. Biggest increase is observed at $CD14^+$ monocyte specific loci. The increase was much stronger in men. B cells specific loci on the average gain accessibility in women and lose accessibility in men with age. (**f**) Enrichment of cell-specific loci among DA peaks. Enrichment p values are calculated using hypergeometric test. Note the activation of innate cells (NK and monocytes) and inactivation of T cells with age. The enrichment p-values were more significant in men compared to women. B cell-specific regions were enriched for closing peaks in men and for opening peaks in women. Grayed out circles are non-significant at a 5% FDR; numbers on circles represent the number of peaks in each category. Source data are provided as a Source Data file for panels c, d, e, f.

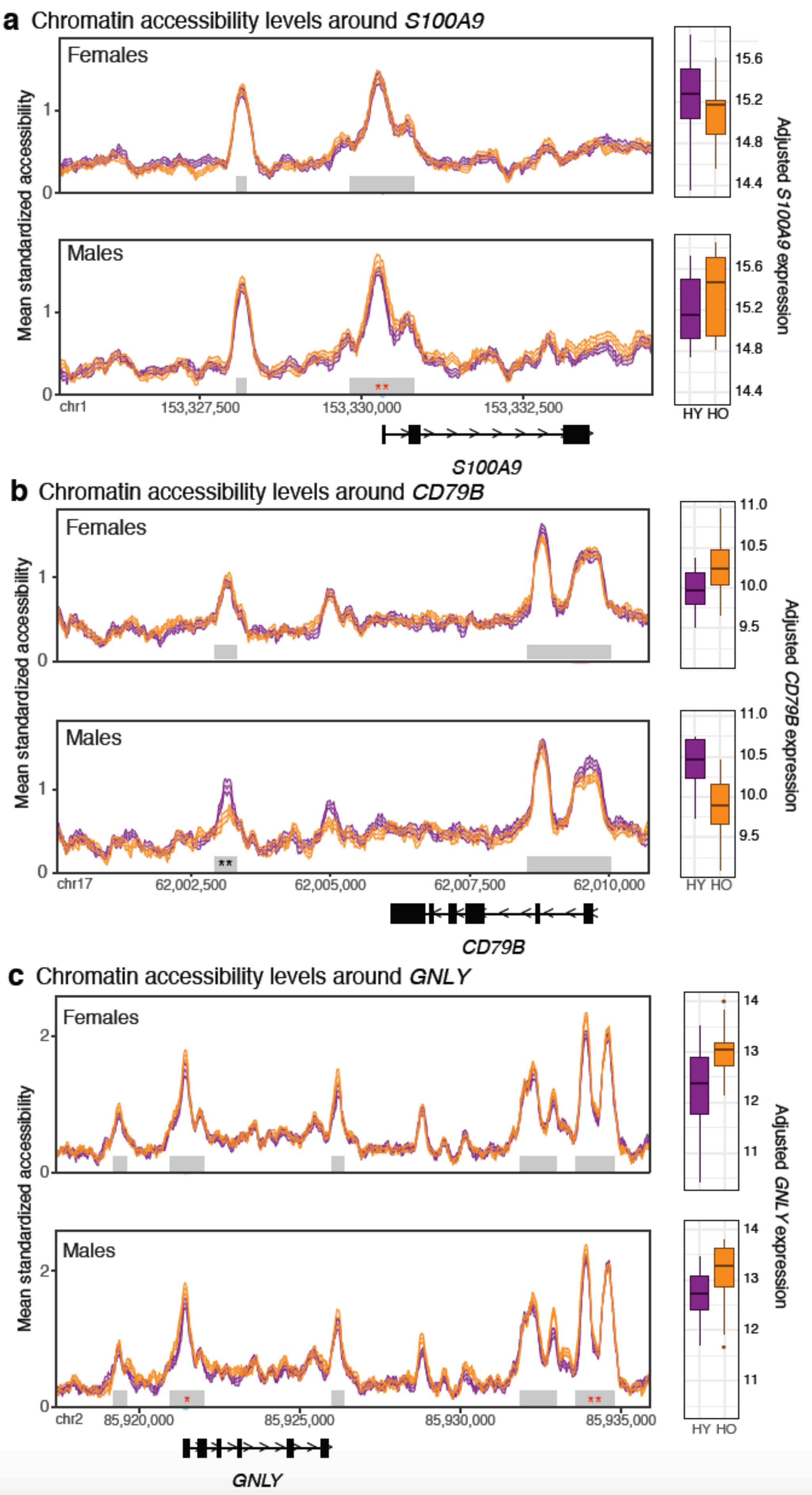
a Chromatin accessibility levels around S100A9
Females
Males
Mean standardized accessibility
chr1
153,327,500
153,330,000
153,332,500
S100A9
15.6
15.2
14.8
14.4
Adjusted S100A9 expression
HY HO
b Chromatin accessibility levels around CD79B
Females
Males
Mean standardized accessibility
chr17
62,002,500
62,005,000
62,007,500
62,010,000
CD79B
11.0
10.5
10.0
9.5
Adjusted CD79B expression
HY HO
c Chromatin accessibility levels around GNLY
Females
Males
Mean standardized accessibility
chr2
85,920,000
85,925,000
85,930,000
85,935,000
GNLY
14
13
12
11
Adjusted GNLY expression
HY HO

Supplementary Figure 3. Chromatin accessibility profiles for example loci. Left: Average chromatin accessibility profiles (grouped by age and sex) around *S100A9* **(a)**, *CD79B* **(b)**, and *GNLY* **(c)** genes in women (top) and men (bottom). The peaks are denoted as gray bars and the ones that are statistically significantly different between young and old samples are marked with stars (red for opening peaks, black for closing peaks). Chromatin accessibility signals for each group includes averages as well as the confidence intervals for the group. Right: Normalized gene expression levels in young (HY) and older (HO) women (top) and men (bottom). HO= healthy old, HY=healthy young.

Figure S4

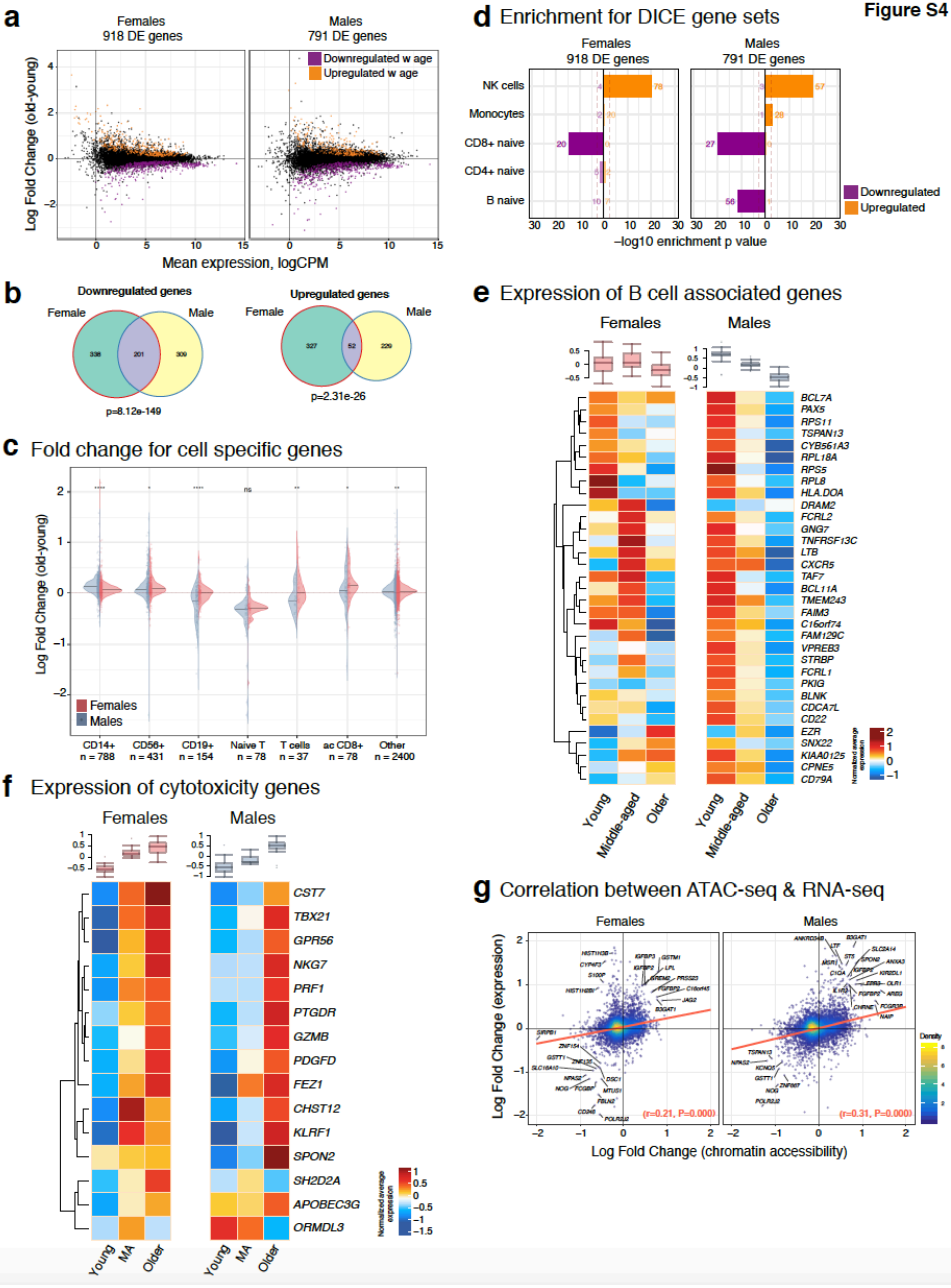
a
Females
918 DE genes
Males
791 DE genes
Downregulated w age
Upregulated w age
Log Fold Change (old-young)
Mean expression, logCPM
b
Downregulated genes
Female
Male
p=8.12e-149
Upregulated genes
p=2.31e-26
c Fold change for cell specific genes
Log Fold Change (old-young)
Females
Males
CD14+ n = 788
CD56+ n = 431
CD19+ n = 154
Naive T n = 78
T cells n = 37
ac CD8+ n = 78
Other n = 2400
d Enrichment for DICE gene sets
NK cells
Monocytes
CD8+ naive
CD4+ naive
B naive
Downregulated
Upregulated
−log10 enrichment p value
e Expression of B cell associated genes
BCL7A
PAX5
RPS11
TSPAN13
CYB561A3
RPL18A
RPS5
RPL8
HLA.DOA
DRAM2
FCRL2
GNG7
TNFRSF13C
LTB
CXCR5
TAF7
BCL11A
TMEM243
FAIM3
C16orf74
FAM129C
VPREB3
STRBP
FCRL1
PKIG
BLNK
CDCA7L
CD22
EZR
SNX22
KIAA0125
CPNE5
CD79A
Young
Middle-aged
Older
f Expression of cytotoxicity genes
CST7
TBX21
GPR56
NKG7
PRF1
PTGDR
GZMB
PDGFD
FEZ1
CHST12
KLRF1
SPON2
SH2D2A
APOBEC3G
ORMDL3
Young
MA
Older
Normalized average expression
g Correlation between ATAC-seq & RNA-seq
Log Fold Change (expression)
Log Fold Change (chromatin accessibility)
(r=0.21, P=0.000)
(r=0.31, P=0.000)
Density

Supplementary Figure 4. Shared and sex-specific transcriptomic signatures. (**a**) MA plot representing log2 fold change (older-young) versus average read count for RNA-seq data in women (left) and men (right). Differentially upregulated (downregulated) genes with age are represented in orange (purple). Differential gene expression was tested using a generalized linear model based on read counts, with significance assessed at a 10% FDR threshold, after using Benjamini-Hochberg P-value adjustment. (**b**) Overlap between differentially expressed (DE) genes in women and men. Note that despite sex differences, overlaps are statistically significant based on Fisher's exact test for p-value calculations. (**c**) Distribution of fold changes for gene expression levels of all cell-specific genes. Positive (negative) fold changes represent upregulation (downregulation) with age. $CD14^+$ monocyte-specific genes are associated with positive fold change in both sexes, higher in men. B cell-specific genes are associated with negative fold changes only in men. (**d**) Enrichment of DE genes using cell-specific gene sets from the DICE database[10]. Naïve T cell genes are enriched in downregulated genes, NK cell genes are enriched in upregulated genes. B cell-specific genes are enriched in downregulated genes only in men. Enrichment p values were calculated using hypergeometric test. (**e**) Average normalized expression levels of B cell specific genes grouped by age group and sex. Box plots on the top summarizes the data from all genes represented in the heatmap. (**f**) Average normalized expression levels of genes in the cytotoxic module grouped by age group and sex. Note the increase in cytotoxic molecules in both sexes. Box plots on the top summarize the data from all genes represented in the heatmap. (**g**) Correlation of age-related chromatin accessibility and gene expression changes based on chromatin accessibility at promoters in women (left) and in men (right). Color schema represent the density of points. Note that age-related remodeling ATAC-seq and RNA-seq data correlates positively and significantly in both sexes. R represents the correlation coefficient. Source data are provided as a Source Data file for panels a, c, d, g.

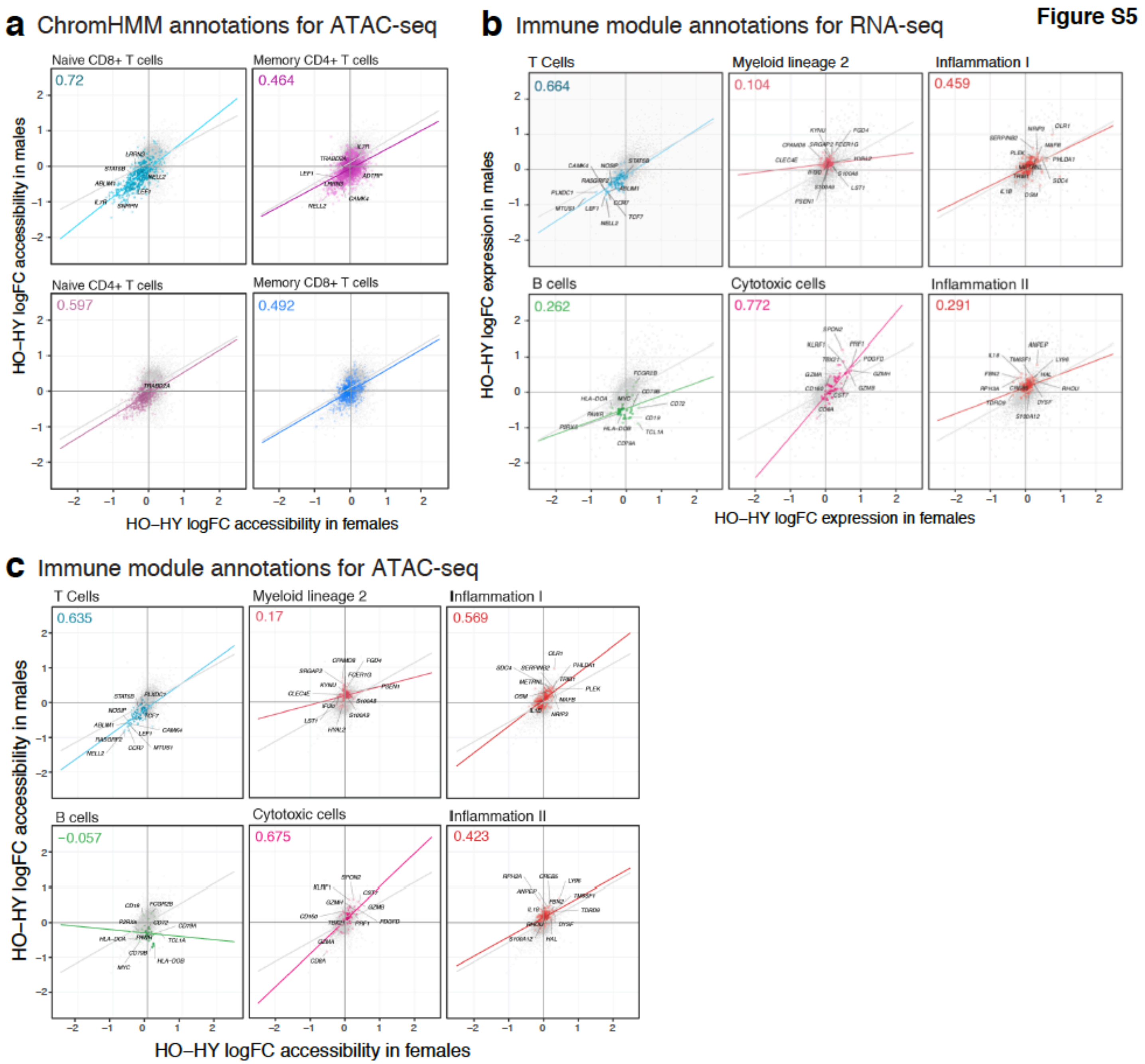

Supplementary Figure 5. Sex-dimorphic changes in monocyte- and B cell-associated loci. (a) Correlation of age-related ATAC-seq changes stratified by T cell subset specific loci from chromHMM states. Values at the top left corner represents the correlation coefficient between sexes only for the genes/loci associated to that cell type. (**b-c**) Correlation of age-related RNA-seq (**b**) and ATAC-seq (**c**) changes stratified by immune modules[22]. Values at the top left corner represents the correlation coefficient between sexes only for the genes/loci associated to that cell type/module. Source data are provided as a Source Data file for panels a, b, c.

Figure S6

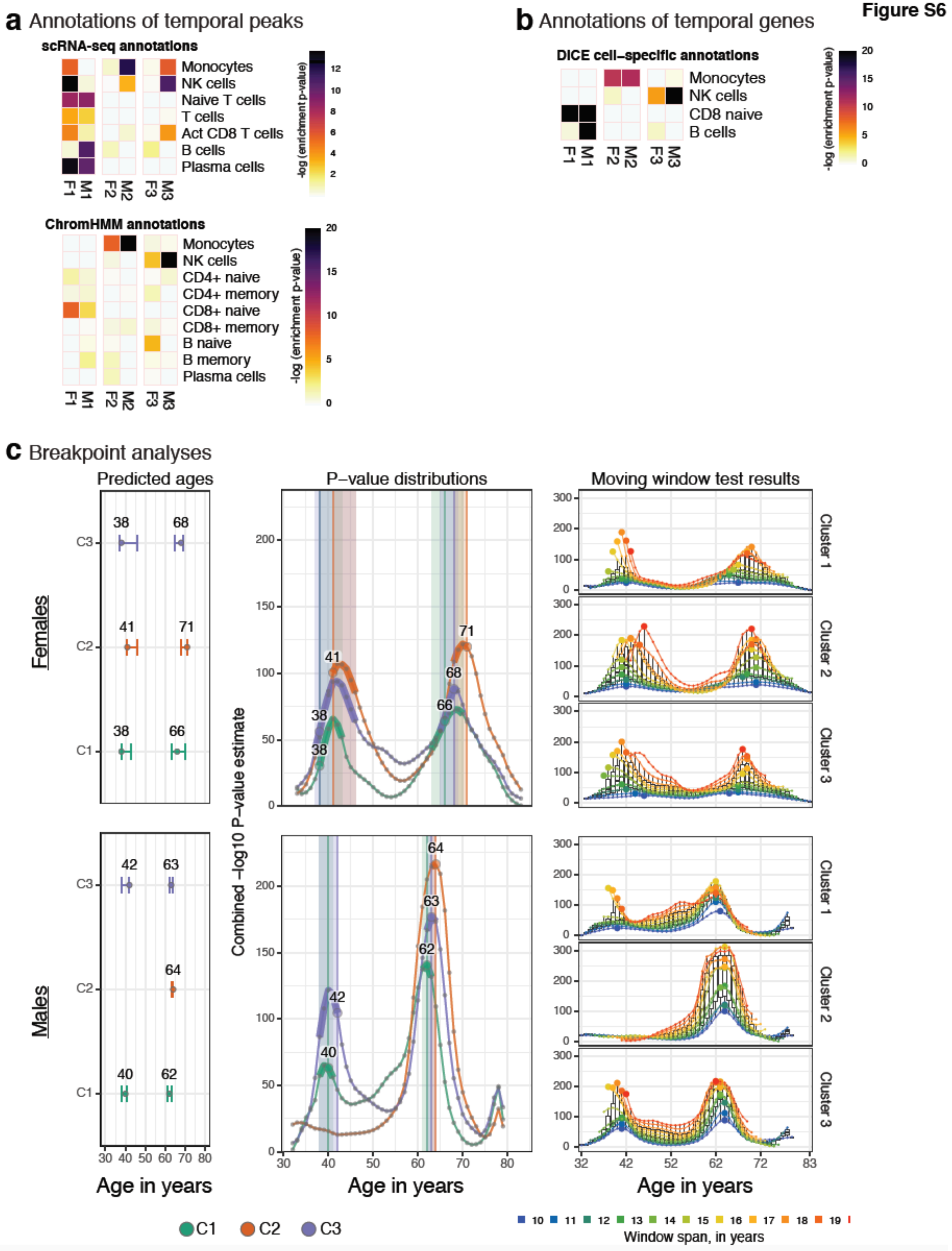

a Annotations of temporal peaks
scRNA-seq annotations
Monocytes
NK cells
Naive T cells
T cells
Act CD8 T cells
B cells
Plasma cells
F1 M1 F2 M2 F3 M3
-log (enrichment p-value)
ChromHMM annotations
Monocytes
NK cells
CD4+ naive
CD4+ memory
CD8+ naive
CD8+ memory
B naive
B memory
Plasma cells
F1 M1 F2 M2 F3 M3
-log (enrichment p-value)
b Annotations of temporal genes
DICE cell-specific annotations
Monocytes
NK cells
CD8 naive
B cells
F1 M1 F2 M2 F3 M3
-log (enrichment p-value)
c Breakpoint analyses
Predicted ages
P-value distributions
Moving window test results
Females
Males
C1
C2
C3
Cluster 1
Cluster 2
Cluster 3
Combined -log10 P-value estimate
Age in years
Age in years
Age in years
C1 C2 C3
10 11 12 13 14 15 16 17 18 19
Window span, in years

Supplementary Figure 6. Epigenome and transcriptome changes over human adult lifespan. (**a**) Annotations of temporal peaks using cell-specific expression (top) and chromatin states (bottom) in women (F1-F3) and in men (M1-M3). Colors represent hypergeometric enrichment test P-values. Single-cell RNA-seq-based enrichments based on nearest-gene annotations of temporal peaks. (**b**) Annotation of temporal genes using cell-specific gene sets from the DICE database[10]. (**c**) Detailed results from breakpoint analysis. Left: age range and median age of estimated breakpoints from each cluster in women (top) and men (bottom); Middle: -log (p-value) of smoothed multi-scale moving window tests. Age range estimates for each cluster are represented as thickened curves and wide vertical bars; Right: -log(p-value) of multi-scale analysis where each curve/color represents a distinct window span for the test, ranging from 10 to 20 years. Stronger evidence of a breakpoint emerges as the same age bracket is detected as significant at multiple window spans. C1, C2, C3 stands for cluster1-3. Source data are provided as a Source Data file for panels a, b.

Figure S7

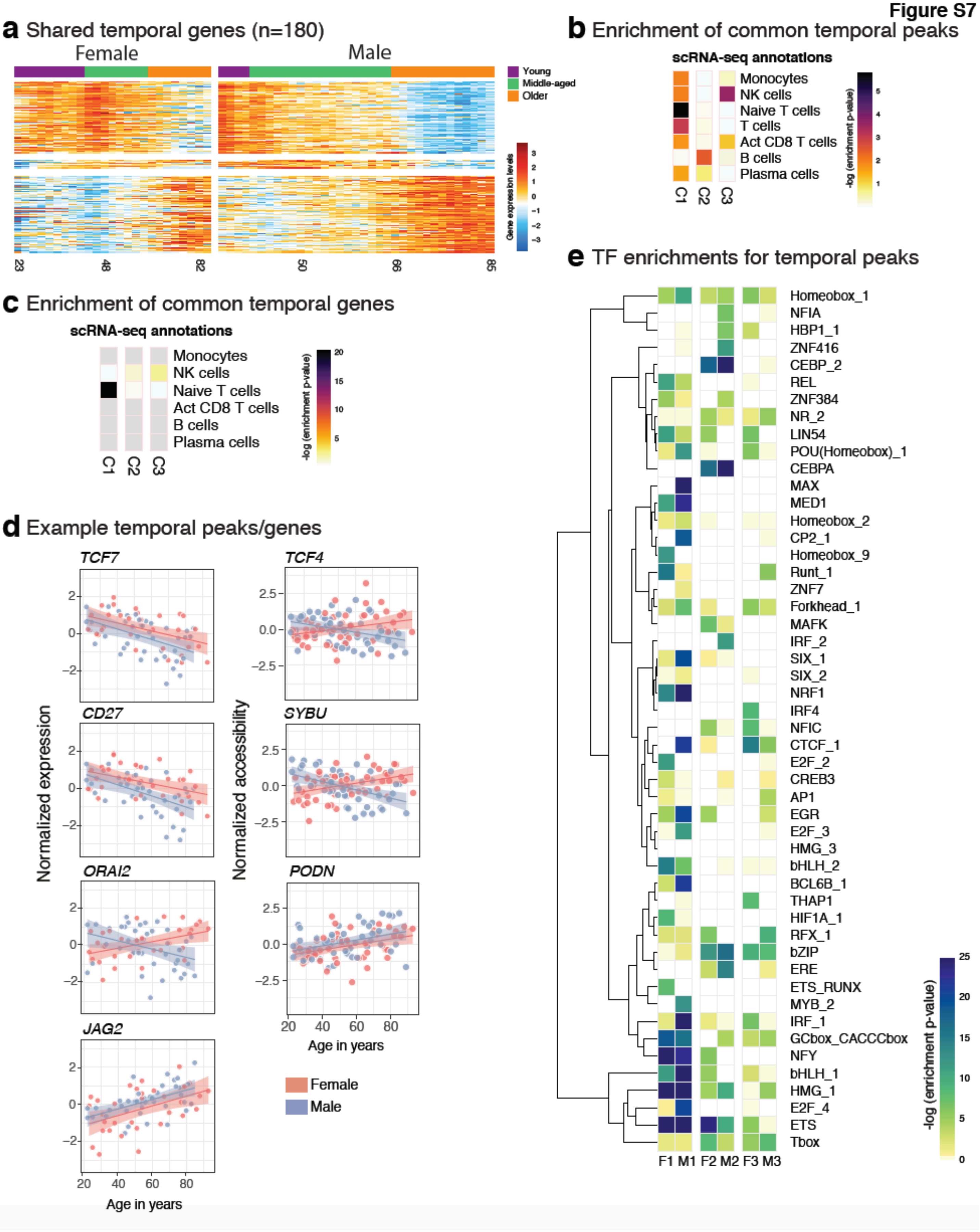

a Shared temporal genes (n=180)
Female
Male
Young
Middle-aged
Older
Gene expression levels
23
48
82
50
66
85
b Enrichment of common temporal peaks
scRNA-seq annotations
Monocytes
NK cells
Naive T cells
T cells
Act CD8 T cells
B cells
Plasma cells
C1
C2
C3
-log (enrichment p-value)
c Enrichment of common temporal genes
scRNA-seq annotations
Monocytes
NK cells
Naive T cells
Act CD8 T cells
B cells
Plasma cells
C1
C2
C3
-log (enrichment p-value)
d Example temporal peaks/genes
TCF7
CD27
ORAI2
JAG2
TCF4
SYBU
PODN
Normalized expression
Normalized accessibility
Age in years
Female
Male
e TF enrichments for temporal peaks
Homeobox_1
NFIA
HBP1_1
ZNF416
CEBP_2
REL
ZNF384
NR_2
LIN54
POU(Homeobox)_1
CEBPA
MAX
MED1
Homeobox_2
CP2_1
Homeobox_9
Runt_1
ZNF7
Forkhead_1
MAFK
IRF_2
SIX_1
SIX_2
NRF1
IRF4
NFIC
CTCF_1
E2F_2
CREB3
AP1
EGR
E2F_3
HMG_3
bHLH_2
BCL6B_1
THAP1
HIF1A_1
RFX_1
bZIP
ERE
ETS_RUNX
MYB_2
IRF_1
GCbox_CACCCbox
NFY
bHLH_1
HMG_1
E2F_4
ETS
Tbox
F1 M1 F2 M2 F3 M3
-log (enrichment p-value)

Supplementary Figure 7. Sex-specific patterns in temporal peaks/genes. (**a**) Heatmap of ARIMA-fitted expression values for genes with a significant chronological trend in both women and men (N=180). Values represent z score normalized gene expression values relative to the row mean. K-means clustering is used to group these peaks into three clusters (C1-C3) using concatenated data from men and women. Color bar on the top represents discrete age groupings as defined in this study (young, middle-aged, older). (**b**) Enrichment of cell-specific gene sets derived from scRNA-seq data among temporal clusters of OCRs, peaks are annotated to their nearest TSS. (**c**) Enrichment of cell-specific gene sets obtained from scRNA-seq among temporal and shared genes. Grey cells in plot indicate that there were insufficient genes in the gene sets to run an enrichment test. (**d**) Example temporal genes/peaks. (**e**) Transcription factor (TF) motif enrichment results for temporal peak clusters obtained separately from men and women. Peaks in each cluster is compared against the peaks in the other two clusters. Motif enrichment analyses carried out based on 1,388 PWMs, grouped into families based on the sequence similarity, and most significant p-value for each family is represented here. Source data are provided as a Source Data file for panels a, b, c.

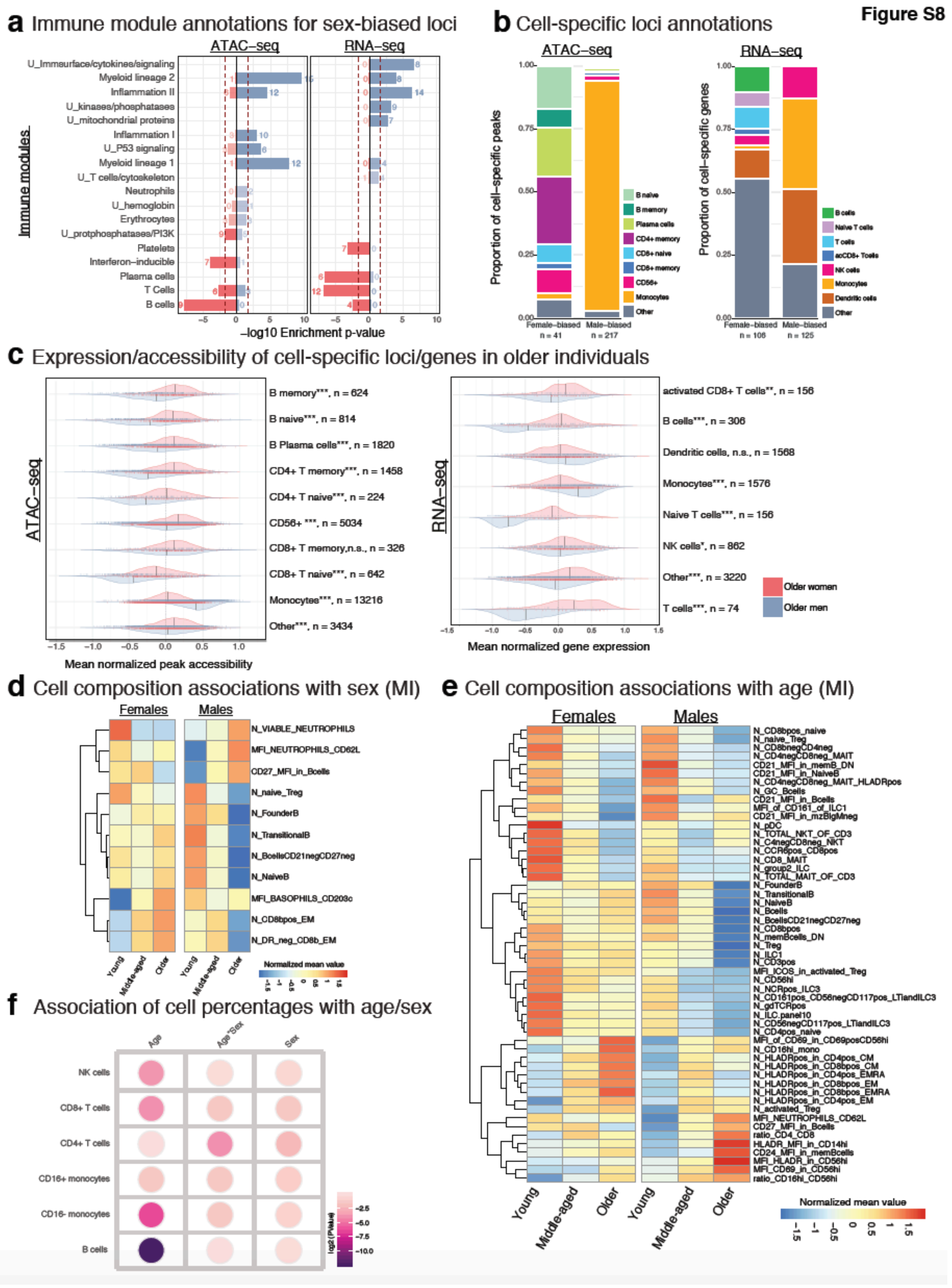
Figure S8
a Immune module annotations for sex-biased loci
ATAC–seq
RNA–seq
Immune modules
U_Immsurface/cytokines/signaling
Myeloid lineage 2
Inflammation II
U_kinases/phosphatases
U_mitochondrial proteins
Inflammation I
U_P53 signaling
Myeloid lineage 1
U_T cells/cytoskeleton
Neutrophils
U_hemoglobin
Erythrocytes
U_protphosphatases/PI3K
Platelets
Interferon–inducible
Plasma cells
T Cells
B cells
–log10 Enrichment p-value
b Cell-specific loci annotations
ATAC–seq
RNA–seq
Proportion of cell-specific peaks
Proportion of cell-specific genes
Female–biased
Male–biased
n = 41
n = 217
n = 106
n = 125
B naive
B memory
Plasma cells
CD4+ memory
CD8+ naive
CD8+ memory
CD56+
Monocytes
Other
B cells
Naive T cells
T cells
acCD8+ Tcells
NK cells
Dendritic cells
c Expression/accessibility of cell-specific loci/genes in older individuals
ATAC–seq
B memory***, n = 624
B naive***, n = 814
B Plasma cells***, n = 1820
CD4+ T memory***, n = 1458
CD4+ T naive***, n = 224
CD56+ ***, n = 5034
CD8+ T memory,n.s., n = 326
CD8+ T naive***, n = 642
Monocytes***, n = 13216
Other***, n = 3434
Mean normalized peak accessibility
RNA–seq
activated CD8+ T cells**, n = 156
B cells***, n = 306
Dendritic cells, n.s., n = 1568
Monocytes***, n = 1576
Naive T cells***, n = 156
NK cells*, n = 862
Other***, n = 3220
T cells***, n = 74
Older women
Older men
Mean normalized gene expression
d Cell composition associations with sex (MI)
Females
Males
N_VIABLE_NEUTROPHILS
MFI_NEUTROPHILS_CD62L
CD27_MFI_in_Bcells
N_naive_Treg
N_FounderB
N_TransitionalB
N_BcellsCD21negCD27neg
N_NaiveB
MFI_BASOPHILS_CD203c
N_CD8bpos_EM
N_DR_neg_CD8b_EM
Young
Middle-aged
Older
Normalized mean value
e Cell composition associations with age (MI)
Females
Males
N_CD8bpos_naive
N_naive_Treg
N_CD8bnegCD4neg
N_CD4negCD8neg_MAIT
CD21_MFI_in_memB_DN
CD21_MFI_in_NaiveB
N_CD4negCD8neg_MAIT_HLADRpos
N_GC_Bcells
CD21_MFI_in_Bcells
MFI_of_CD161_of_ILC1
CD21_MFI_in_mzBIgMneg
N_pDC
N_TOTAL_NKT_OF_CD3
N_C4negCD8neg_NKT
N_CCR6pos_CD8pos
N_CD8_MAIT
N_group2_ILC
N_TOTAL_MAIT_OF_CD3
N_FounderB
N_TransitionalB
N_NaiveB
N_Bcells
N_BcellsCD21negCD27neg
N_CD8bpos
N_memBcells_DN
N_Treg
N_ILC1
N_CD3pos
MFI_ICOS_in_activated_Treg
N_CD56hi
N_NCRpos_ILC3
N_CD161pos_CD56negCD117pos_LTiandILC3
N_gdTCRpos
N_ILC.panel10
N_CD56negCD117pos_LTiandILC3
N_CD4pos_naive
MFI_of_CD69_in_CD69posCD56hi
N_CD16hi_mono
N_HLADRpos_in_CD4pos_CM
N_HLADRpos_in_CD8bpos_CM
N_HLADRpos_in_CD4pos_EMRA
N_HLADRpos_in_CD8bpos_EM
N_HLADRpos_in_CD8bpos_EMRA
N_HLADRpos_in_CD4pos_EM
N_activated_Treg
MFI_NEUTROPHILS_CD62L
CD27_MFI_in_Bcells
ratio_CD4_CD8
HLADR_MFI_in_CD14hi
CD24_MFI_in_memBcells
MFI_HLADR_in_CD56hi
MFI_CD69_in_CD56hi
ratio_CD16hi_CD56hi
Young
Middle-aged
Older
Normalized mean value
f Association of cell percentages with age/sex
Age
Age*Sex
Sex
NK cells
CD8+ T cells
CD4+ T cells
CD16+ monocytes
CD16- monocytes
B cells
log2 (PValue)

Supplementary Figure 8. Genomic differences between sexes at different ages. (**a**) Enrichment of sex-biased peaks/genes in older individuals using immune modules[22]. Note the male bias toward increased accessibility/expression for myeloid lineage and inflammation modules and the female bias toward T and B cell related modules. P-values are based on hypergeometric enrichment tests. Numbers on barplots represent the number of gene hits for each module. (**b**) Distribution of cell-specific loci among sex-biased peaks (left) and sex-biased genes (right). Note the enrichment of monocyte specific loci among male biased loci/genes. (**c**) Distribution of chromatin accessibility (left) and gene expression levels (right) in older men (blue) and older women (pink) for cell-specific genes. Stars next to the cell type name represent whether there is a significant difference between distributions in men and women. N represents the total number of cell-specific loci/genes used in each analysis. (**d**) Flow cytometry data from Milieu Intérieur Consortium[12] (n=892) re-analyzed using generalized linear models (GLMs) to associate cell counts or MFI values to sex. Rows represent the measurements that has been significantly associated to sex (FDR 5%). Values in heatmap represent the average value across individuals stratified based on sex and age group. Note the male-specific decline in different B cell populations after 65 years old, in agreement with our cohort. (**e**) Flow cytometry data from Milieu Intérieur Consortium[12] (n=892) re-analyzed using generalized linear models (GLMs) to associate cell counts or MFI values to age. Rows represent the measurements that has been significantly associated to age (FDR 5%). Values in heatmap represent the average value across individuals stratified based on sex and age group. (**f**) Mass cytometry data from Whiting *et. al.*[29] re-analyzed using linear models to associate cell percentages to age and sex. Colors represent the association p-value. Note that B cells are the most significantly associated to age in this cohort. Source data are provided as a Source Data file for panels a, b, c.
