# Supplementary material for: Sexual-dimorphism in human immune system aging
Source: Nat Commun. 2020 Feb 6;11:751. doi: 10.1038/s41467-020-14396-9 (PMC7005316; doi:10.1038/s41467-020-14396-9)
Supplement: Supplementary file 18 — Reporting Summary [file 41467_2020_14396_MOESM18_ESM.pdf]

nature research

Corresponding author(s): Duygu Ucar

Last updated by author(s): Dec 23, 2019

Reporting Summary

Nature Research wishes to improve the reproducibility of the work that we publish. This form provides structure for consistency and transparency in reporting. For further information on Nature Research policies, see Authors & Referees and the Editorial Policy Checklist.

Statistics

For all statistical analyses, confirm that the following items are present in the figure legend, table legend, main text, or Methods section.

n/a	Confirmed	
☐	☒	The exact sample size (*n*) for each experimental group/condition, given as a discrete number and unit of measurement
☐	☒	A statement on whether measurements were taken from distinct samples or whether the same sample was measured repeatedly
☐	☒	The statistical test(s) used AND whether they are one- or two-sided<br>*Only common tests should be described solely by name; describe more complex techniques in the Methods section.*
☐	☒	A description of all covariates tested
☐	☒	A description of any assumptions or corrections, such as tests of normality and adjustment for multiple comparisons
☐	☒	A full description of the statistical parameters including central tendency (e.g. means) or other basic estimates (e.g. regression coefficient) AND variation (e.g. standard deviation) or associated estimates of uncertainty (e.g. confidence intervals)
☐	☒	For null hypothesis testing, the test statistic (e.g. *F*, *t*, *r*) with confidence intervals, effect sizes, degrees of freedom and *P* value noted<br>*Give P values as exact values whenever suitable.*
☒	☐	For Bayesian analysis, information on the choice of priors and Markov chain Monte Carlo settings
☒	☐	For hierarchical and complex designs, identification of the appropriate level for tests and full reporting of outcomes
☒	☐	Estimates of effect sizes (e.g. Cohen's *d*, Pearson's *r*), indicating how they were calculated

Our web collection on statistics for biologists contains articles on many of the points above.

Software and code

Policy information about availability of computer code

Data collection	No software was used.
Data analysis	R software is used for data analyses. The code is available at (https://github.com/UcarLab/SexDimorphismNatureCommunications). The R Shiny app is publicly shared at (https://immune-aging.jax.org/). Code for the R Shiny app is available at https://github.com/TheJacksonLaboratory/Ucar_Aging_Shiny.

For manuscripts utilizing custom algorithms or software that are central to the research but not yet described in published literature, software must be made available to editors/reviewers. We strongly encourage code deposition in a community repository (e.g. GitHub). See the Nature Research guidelines for submitting code & software for further information.

Data

Policy information about availability of data

All manuscripts must include a data availability statement. This statement should provide the following information, where applicable:

- Accession codes, unique identifiers, or web links for publicly available datasets
- A list of figures that have associated raw data
- A description of any restrictions on data availability

The data generated as part of this study is controlled access. All fastq files for ATAC-seq and RNA-seq samples (including EGA samples) are deposited to dbGAP (phs001934.v1.p1). Flow cytometry data is provided as Supplementary Table 4.

Field-specific reporting

Please select the one below that is the best fit for your research. If you are not sure, read the appropriate sections before making your selection.

☒ Life sciences ☐ Behavioural & social sciences ☐ Ecological, evolutionary & environmental sciences

For a reference copy of the document with all sections, see nature.com/documents/nr-reporting-summary-flat.pdf

Life sciences study design

All studies must disclose on these points even when the disclosure is negative.

Sample size	Data from 172 human subjects were provided as part of this study, this includes PBMCs analyzed with RNA-seq (n=75), ATAC-seq (n=120), flow cytometry (n=129).
Data exclusions	Some ATAC-seq samples are excluded based on QC explained in details in methods.
Replication	Most of our findings were reproduced in other flow cytometry datasets. Specifically we analyzed data from three other studies and reported those findings.
Randomization	There was no randomization of cohorts. Allocations were done based on age groups of participants in the parent study.
Blinding	No blinding. Samples were collected from a healthy cohort of adults.

Reporting for specific materials, systems and methods

We require information from authors about some types of materials, experimental systems and methods used in many studies. Here, indicate whether each material, system or method listed is relevant to your study. If you are not sure if a list item applies to your research, read the appropriate section before selecting a response.

Materials & experimental systems

n/a	Involved in the study	
☐	☒	Antibodies
☒	☐	Eukaryotic cell lines
☒	☐	Palaeontology
☒	☐	Animals and other organisms
☐	☒	Human research participants
☐	☒	Clinical data

Methods

n/a	Involved in the study	
☒	☐	ChIP-seq
☐	☒	Flow cytometry
☒	☐	MRI-based neuroimaging

Antibodies

Antibodies used	CD3 (UCHT1) Biolegend-1:100 (cat# 300436)<br>CD4 (RPA-T4) Biolegend 1:80 (cat# 558116)<br>CD8 (SCFI21Thy2D3) Beckman Coulter 1:80 (cat# 6604728)<br>CD45RA (HI100) BD biosciences 1:80 (cat# 560674)<br>CD19 (HIB19) BD biosciences 1:100 (cat#-555415)<br>CD14 (MSE2) BD biosciences 1:80 (cat# 557923)<br>CCR7 (150503) BD biosciences 1:20 (cat# 561271)
Validation	Antibodies are used according to manufacturers' instructions.

Human research participants

Policy information about studies involving human research participants

Population characteristics	Information on the subjects are provided in Supplementary Table 1, which includes age, race, sex, ethnicity, BMI, CMV positivity, frailty index.
Recruitment	Following informed consent, blood samples were obtained from 172 healthy volunteers residing in the Greater Hartford, CT, USA region recruited by the UConn Center on Aging Recruitment and Community Outreach Research Core (http://health.uconn.edu/aging/research/research-cores/). For older adults 65 years and older, recruitment criteria were selected to identify individuals who are experiencing “usual healthy” aging and are thus representative of the average or typical normal health status of the local population within the corresponding age groups5 Selecting this type of cohort is in keeping with the 2019 NIH Policy on Inclusion Across the Lifespan (NOT-98-024)40, increasing the generalizability of our studies and the likelihood that these findings

can be translated to the general population41. Subjects were carefully screened in order to exclude potentially confounding diseases and medications, as well as frailty. Individuals who reported chronic or recent (i.e., within two weeks) infections were also excluded. Subjects were deemed ineligible if they reported a history of diseases such as congestive heart failure, ischemic heart disease, myocarditis, congenital abnormalities, Paget's disease, kidney disease, diabetes requiring insulin, chronic obstructive lung disease, emphysema, and asthma. Subjects were also excluded if undergoing active cancer treatment, prednisone above 10 mg day, other immunosuppressive drugs, any medications for rheumatoid arthritis other than NSAIDs or if they had received antibiotics in the previous 6 months. Beyond these steps to exclude specific chronic conditions we also undertook further additional efforts to exclude older adults with any significant frailty. Since declines in self-reported physical performance are highly predictive of frailty, subsequent disability and mortality42, all subjects were also questioned as to their ability to walk ¼ mile (or 2-3 city blocks). For those who self-reported an inability to walk ¼ mile42, the "Timed Up and Go" (TUG) test was performed and measured as the time taken to stand up from the sitting position, walk 10 feet and return to sitting in the chair43. Scoring TUG > 10 sec was considered an indication of increased frailty and resulted in exclusion from the study44.

Ethics oversight: The study was conducted following approval by the Institutional Review Board of UConn Health Center (IRB Number: 14-194J-3).

Note that full information on the approval of the study protocol must also be provided in the manuscript.

Clinical data

Policy information about clinical studies

All manuscripts should comply with the ICMJE guidelines for publication of clinical research and a completed CONSORT checklist must be included with all submissions.

Clinical trial registration	NA.
Study protocol	This is performed as a substudy to an on-going protocol, thus protocol will not be shared.
Data collection	Community dwelling adults recruited through UCHC Aging Center. Samples are collected over period of June 2014 and February 2018.
Outcomes	This is a basic sciences study and outcome measures are genomic readouts.

Flow Cytometry

Plots

Confirm that:

- [ ] The axis labels state the marker and fluorochrome used (e.g. CD4-FITC).
- [ ] The axis scales are clearly visible. Include numbers along axes only for bottom left plot of group (a 'group' is an analysis of identical markers).
- [ ] All plots are contour plots with outliers or pseudocolor plots.
- [x] A numerical value for number of cells or percentage (with statistics) is provided.

Methodology

Sample preparation	PBMCs were isolated from fresh whole blood using Ficoll-Paque Plus (GE) density gradient centrifugation.
Instrument	BD Fortessa X20.
Software	The stained cells were acquired with BD FACS Diva and analyzed with FlowJo software (TreeStar).
Cell population abundance	Only cell compositions of PBMCs are studied here. Cells are not sorted.
Gating strategy	Gated mononuclear cells, singlets, CD3 vs. CD19 gating, CD14 gated on CD3- CD19- cells. Sup Figure 2a.

- [x] Tick this box to confirm that a figure exemplifying the gating strategy is provided in the Supplementary Information.
